# The changes of corneal biomechanical properties with long-term treatment of prostaglandin analogue measured by Corvis ST

**DOI:** 10.1186/s12886-020-01693-6

**Published:** 2020-10-20

**Authors:** Na Wu, Yuhong Chen, Yaping Yang, Xinghuai Sun

**Affiliations:** 1 Eye Institute and Department of Ophthalmology, Eye & ENT Hospital, Fudan University, Shanghai, 200031 China; 2 NHC Key Laboratory of Myopia (Fudan University); Key Laboratoy of Myopia, Chinese Academy of Medical Sciences, Shanghai, 200031 China; 3 Shanghai Key Laboratoy of Visual Impairment and Restoration, Shanghai, 200031 China; 4 State Key Laboratory of Medical Neurobiology, Institutes of Brain Science and Collaborative Innovation Center for Brain Science, Fudan University, Shanghai, 200032 China

**Keywords:** Prostaglandin analogue, Corneal biomechanical properties, Glaucoma, Corvis ST

## Abstract

**Background:**

To investigate the corneal biomechanical changes in primary open angle glaucoma (POAG) patients treated with long-term prostaglandin analogue (PGA).

**Methods:**

One hundred eleven newly diagnosed POAG patients, including 43 high tension glaucoma (HTG) and 68 normal tension glaucoma (NTG), were measured by Corvis ST to obtain intraocular pressure (IOP), central corneal thickness (CCT) and corneal biomechanical parameters at baseline and at each follow-up visit after initiation of PGA treatment. The follow-up measurements were analyzed by the generalized estimate equation model with an exchangeable correlation structure. Restricted cubic spline was employed to estimate the dose–response relation between follow-up time and corneal biomechanics.

**Results:**

The mean follow-up time was 10.3 ± 7.02 months. Deformation amplitude (β = -0.0015, *P* = 0.016), the first applanation velocity (AV1, β = -0.0004, *P* = 0.00058) decreased and the first applanation time (AT1, β = 0.0089, *P* < 0.000001) increased statistically significantly with PGA therapy over time after adjusting for age, gender, axial length, corneal curvature, IOP and CCT. In addition, AT1 was lower (7.2950 ± 0.2707 in NTG and 7.5889 ± 0.2873 in HTG, *P* = 0.00011) and AV1 was greater (0.1478 ± 0.0187 in NTG and 0.1314 ± 0.0191 in HTG, *P* = 0.00002) in NTG than in HTG after adjusting for confounding factors.

**Conclusions:**

Chronic use of PGA probably influences the corneal biomechanical properties directly, which is to make cornea less deformable. Besides, corneas in NTG tended to be more deformable compared to those in HTG with long-term treatment of PGA.

## Background

Corneal biomechanical properties have recently attracted increasing attention for its involvement in glaucoma and in other ocular diseases, such as keratoconus [1]. The biomechanical properties of cornea may determine its deformation response to applanation, which influences the intraocular pressure (IOP) measurement [2, 3]. In addition, it has been speculated that corneal biomechanics may represent structural vulnerabilities of the whole eyeball to the development of glaucoma [4–6]. Thus, understanding corneal biomechanics is helpful to improve the diagnosis and management of glaucoma [2, 3, 7].

Primary open angle glaucoma (POAG) is the most common type of glaucoma worldwide. Prostaglandin analogue (PGA) is the first-line hypotensive medication for POAG currently. Long-term use of PGA has been reported to decrease the central corneal thickness (CCT) due to activation of corneal stromal matrix metalloproteinases (MMPs) [8, 9]. Experimental studies also indicate that PGA causes morphological and biochemical changes of corneal stroma [10]. These changes imply that corneal biomechanical properties could possibly be affected by PGA.

Most of the available studies about corneal biomechanics were conducted by using the Ocular Response Analyzer (ORA) [11]. ORA is the first developed device, which is able to provide an in vivo measurement of corneal viscoelastic parameter called corneal hysteresis (CH) [12]. CH is shown to be decreased in glaucomatous eyes compared to that in normal controls [13] and associated with visual field progression in glaucomatous eyes [7].

The Corneal Visualization Scheimpflug Technology (Corvis ST) is a newly available non-touch instrument that provides precise, repeatable, and reproducible measurements of corneal biomechanics in vivo [11, 14]. The Corvis ST uses an ultra-high-speed Scheimpflug camera to record corneal deformation in real time and provides direct information on corneal biomechanical behavior [15] and could potentially provide more information on corneal biomechanics than ORA [16].

Our previous study conducted by Corvis ST observed significant differences in biomechanical parameters between treatment naïve POAG patients and patients with long-term therapy of PGA, even after adjusting for other confounding factors that might influence biomechanics, such as IOP and CCT [17]. The results suggested that PGA might have a potential effect on corneal biomechanical properties in addition to its indirect effect owning to IOP decrease and CCT reduction [17]. However, due to the cross-sectional design, the influence of PGA on corneal biomechanics remains unanswered. In this study, we prospectively followed up newly diagnosed POAG patients under PGA treatment to observe the changes of corneal biomechanical properties over time.

## Methods

This research was approved by the Medical Ethics Committee of Eye and ENT Hospital of Fudan University, and adhered to the Declaration of Helsinki. Written informed consents were obtained from all of the participants.

Patients who were newly diagnosed with POAG were recruited consecutively at Eye and ENT Hospital of Fudan University. POAG was diagnosed with the criteria as follows: an open anterior chamber angle on gonioscopy examination, characteristic glaucomatous optic disc changes (thinning of the optic disc rim and/or enlargement of the optic disc cupping) with corresponding visual field defects: (1) glaucoma hemifield test values outside the normal limits; or (2) three or more abnormal points with a probability of being normal of *P* < 5%, of which at least one point has a pattern deviation of *P* < 1%; or (3) a pattern standard deviation of *P* < 5%. In addition, patients were diagnosed with high tension glaucoma (HTG) when they had at least one measurement of IOP > 21 mmHg with a Goldmann applanation tonometer. Patients diagnosed as normal tension glaucoma (NTG) had an IOP ≤ 21 mmHg at all time points on a 24-h IOP variation test. Patients who received continuous therapy of only one type of PGA medication were enrolled in the study. Exclusion criteria included any coexisted ocular conditions such as corneal diseases, previous history of ocular laser or surgical treatment, which might affect corneal biomechanics. Secondary glaucoma, such as trauma, uveitis and steroid induced glaucoma, was excluded as well.

All participants underwent a comprehensive ophthalmologic examination when they were first enrolled in the study, including best corrected visual acuity, slit-lamp biomicroscopy, fundus evaluation with a 90D lens, gonioscopy examination, automated perimetry (Humphrey Field Analyzer, Carl Zeiss, USA) and the retinal nerve fiber layer thickness on optical coherence tomography (OCT) (Optovue RTVue OCT, USA). Axial length and corneal curvature were measured by IOL Master (Zeiss, Germany).

The Corvis ST (Oculus, GmbH, Wetzlar, Germany) not only measures IOP, biomechanically corrected IOP (bIOP), CCT, but also provides parameters of corneal biomechanical properties by exerting an air impulse on the cornea. Under the pressure, the cornea bends inward from the resting state to the first applanation point and continues to move until reaches the maximum deformation state, namely highest concavity (HC). When the pressure of air puff decreases, the cornea moves outward and passes the second applanation point before arriving at its resting state [11, 14]. The biomechanical parameters used in this study were: 1) time from the resting state to the first and second applanations (AT1 and AT2, respectively); 2) corneal velocity during the first and second applantions (AV1 and AV2, respectively); 3) highest concavity time (HC-time) and maximum deformation amplitude (DA) from the resting state to the HC at the corneal apex; peak distance between corneal bending points (HC-PD) and radius of curvature (HC-radius) at HC [11, 14, 18].

Patients were examined by Corvis ST at baseline and at each follow-up visit. Both eyes were included if they were eligible. Single eyes were selected on 7 patients because the other ones could not obtain reliable measurements. It should be noted that only Corvis ST measurements of post-treatment of PGA were used for statistical analysis.

Statistical analyses were performed with R 3.3 (https://www.R-project.org).

Descriptive statistical results were presented as the mean ± standard deviation (SD). The repeated follow-up measurements were analyzed by the generalized estimate equation model with an exchangeable correlation structure. Restricted cubic spline was employed to estimate the dose–response relation between follow-up time and IOP, CCT and corneal biomechanical parameters with adjustment. Independent samples *t* test and chi-square test were used for comparison of demographic (age and gender) and clinical (axial length and corneal curvature) measurements between NTG and HTG groups. It was considered statistically significant if the *P* value was less than 0.05.

## Results

**Fig. 1:**
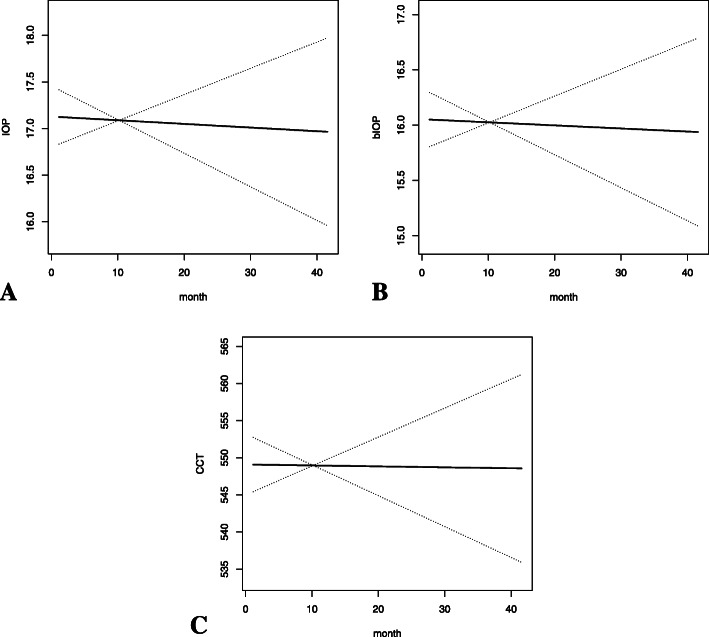
The changes of IOP (**a**), bIOP (**b**) and CCT (**c**) in POAG patients under PGA treatment over time

The examined group consisted of 111 newly diagnosed POAG patients, including 43 female and 68 male with a mean age of 48.04 ± 14.14 years (22—82 years). Together, 215 eyes were examined (both eyes from 104 subjects and 7 eyes from 7 subjects). The mean follow-up time was 10.3 ± 7.02 months (1.15—41.57 months). Subjects had an average of 2.53 ± 1.42 (1–7) times of follow-up visits in the study. The mean axial length and corneal curvature of the total subjects were 25.6 ± 1.77 mm and 7.75 ± 0.24 mm, respectively (Table 1). A slight but statistically significant decrease in IOP (β = -0.0596, *P* = 0.000047), bIOP (β = -0.0369, *P* = 0.0029) and CCT (β = -0.6639, *P* < 0.000001) was observed during the period of PGA usage (Table 2, Fig. 1).

**Table 1:** Demographic, clinical and diagnostic characteristics of 111 newly diagnosed primary open angle glaucoma patients participating in the study

Category	
Age, years	48.04 ± 14.14 (22–82)
Gender	
Female	43 (38.7%)
Male	68 (61.3%)
Follow-up period, months	10.3 ± 7.02 (1.15—41.57)
Follow-up times	2.53 ± 1.42 (1–7)
Diagnosis	
High tension glaucoma (HTG)	43 (38.7%)
Normal tension glaucoma (NTG)	68 (61.3%)
Axial length, mm	25.6 ± 1.77
Corneal curvature, mm	7.75 ± 0.24

**Table 2:** The changes of IOP, bIOP and CCT in POAG patients under PGA treatment over time

	β	95% CI	*P* value
IOP	-0.0596	-0.0883 to -0.0309	**0.000047**
bIOP	-0.0369	-0.0612 to -0.0126	**0.0029**
CCT	-0.6639	-0.8048 to -0.5230	** < 0.000001**

*IOP* intraocular pressure, *bIOP* biomechanically corrected intraocular pressure, *CCT* central corneal thickness, *POAG* primary open angle glaucoma, *PGA* prostaglandin analogue

**Fig. 2:**
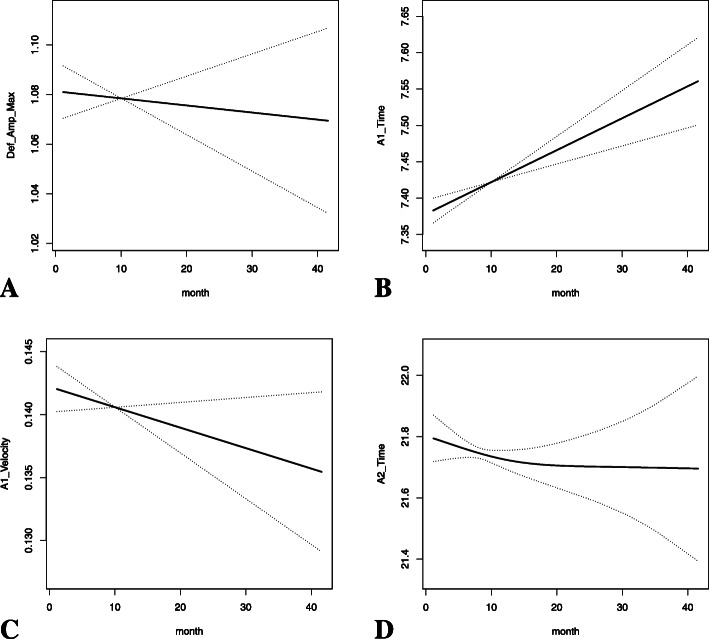
The changes of DA (**a**), AT1 (**b**), AV1 (**c**) and AT2 (**d**) in POAG patients under PGA treatment over time

Generalized estimate equation model with an exchangeable correlation structure was used to observe the corneal biomechanical changes with PGA treatment over time. After adjusting for age, gender, axial length, corneal curvature, IOP and CCT, AT1 increased (β = 0.0089, *P* < 0.000001), whereas DA and AV1 decreased statistically significantly over time (β = -0.0015, *P* = 0.016 for DA; β = -0.0004, *P* = 0.00058 for AV1) (Table 3, Fig. 2). Other corneal biomechanical parameters showed no statistically significant changes over time (Table 3).

**Table 3:** The changes of corneal biomechanical parameters in POAG patients under PGA treatment over time with adjustment for age, gender, axial length, corneal curvature, IOP and CCT

Corneal biomechanical parameters	After adjustment	
	β	*P* value
DA	-0.0015	**0.016**
AT1	0.0089	** < 0.000001**
AV1	-0.0004	**0.00058**
AT2	-0.0059	0.139
AV2	0.0003	0.364
HC-time	-0.0044	0.272
HC-PD	-0.0023	0.196
HC-radius	-0.0076	0.346

*POAG* primary open angle glaucoma, *PGA* prostaglandin analogue, *IOP* intraocular pressure, *CCT* central corneal thickness, *DA* deformation amplitude, *AT1* the first applanation time, *AV1* the first applanation velocity, *AT2* the second applanation time, *AV2* the second applanation velocity, *HC* the highest concavity, *PD* peak distance

**Fig. 3:**
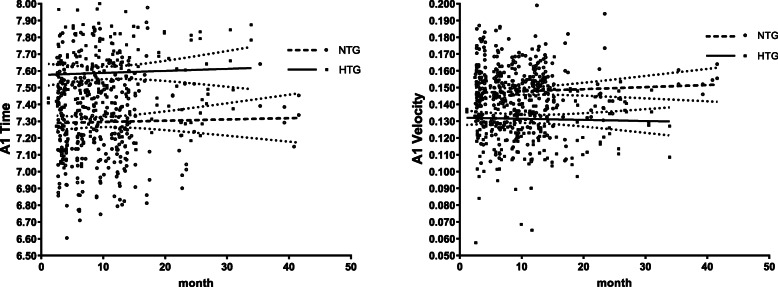
The changes of AT1 (**a**) and AV1 (**b**) in NTG and HTG patients under PGA treatment over time

In addition, we analyzed the differences of corneal biomechanical parameters between NTG and HTG groups under PGA treatment over time. Among the 111 POAG patients, 68 were NTG patients and the other 43 were HTG (Table 1). There were no statistically significant differences in age, gender, axial length and corneal curvature between these two groups (all *P* > 0.05, Table 4). IOP, bIOP and CCT of post-treatment of PGA in NTG group were all statistically significantly lower than those in HTG group (all *P* < 0.0001, Table 4). After correcting for age, gender, axial length, corneal curvature, IOP and CCT, AT1 was lower (7.2950 ± 0.2707 in NTG and 7.5889 ± 0.2873 in HTG, *P* = 0.00011, Fig. 3a) and AV1 was higher (0.1478 ± 0.0187 in NTG and 0.1314 ± 0.0191 in HTG, *P* = 0.00002, Fig. 3b) in NTG group compared to that in HTG group with statistically significant differences (Table 5).

**Table 4:** Differences in demographic and clinical characteristics between NTG and HTG groups

	NTG (*n* = 68)	HTG (*n* = 43)	*P* value
Age (male)	49.15 ± 14.51	46.28 ± 13.50	0.292
Gender	41 (60.3%)	27 (62.8%)	0.843
Axial length	25.52 ± 1.81	25.74 ± 1.70	0.374
Corneal curvature	7.76 ± 0.22	7.75 ± 0.27	0.826
IOP	16.05 ± 2.36	18.47 ± 2.36	** < 0.0001**
bIOP	15.37 ± 2.09	16.90 ± 2.10	** < 0.0001**
CCT	537.44 ± 32.13	564.35 ± 27.96	** < 0.0001**

*NTG* normal tension glaucoma, *HTG* high tension glaucoma, *IOP* intraocular pressure, *bIOP* biomechanically corrected intraocular pressure, *CCT* central corneal thickness

**Table 5:** Differences in corneal biomechanical parameters between NTG and HTG groups under PGA treatment over time after adjusting for age, gender, axial length, corneal curvature, IOP and CCT

	NTG (*n* = 68)	HTG (*n* = 43)	*P* value
DA	1.1070 ± 0.1115	1.0425 ± 0.1248	0.681
AT1	7.2950 ± 0.2707	7.5889 ± 0.2873	**0.00011**
AV1	0.1478 ± 0.0187	0.1314 ± 0.0191	**0.00002**
AT2	21.8646 ± 0.6164	21.6138 ± 0.5827	0.173
AV2	-0.2682 ± 0.0580	-0.2551 ± 0.0426	0.709
HC-time	16.8531 ± 0.5389	16.8520 ± 0.8962	0.477
HC-PD	5.0539 ± 0.2704	4.8712 ± 0.3023	0.241
HC-radius	6.5643 ± 0.9992	6.8861 ± 2.5276	0.428

*NTG* normal tension glaucoma, *HTG* high tension glaucoma, *PGA* prostaglandin analogue, *IOP* intraocular pressure, *CCT* central corneal thickness, *DA* deformation amplitude, *AT1* the first applanation time, *AV1* the first applanation velocity, *AT2* the second applanation time, *AV2* the second applanation velocity, *HC* the highest concavity, *PD* peak distance

## Discussion

PGA is the most widely used first-line therapeutic medication for POAG due to its highest effectiveness in decreasing IOP [19] and reducing circadian IOP fluctuations, as well as its convenient dosing and tolerable side effects [20]. However, PGA was found to have some other effects, such as decreasing the thickness of central cornea. Our results showed CCT declined under treatment of PGA over time, which was in accordance with previous studies [21, 22], implying a possible time cumulative effect of PGA on cornea tissue. The mechanisms of CCT reduction are possibly due to the MMPs up-regulation and extracellular matrix degradation stimulated by PGA in the cornea [8, 9, 23].

The cornea is considered to have both viscous and elastic properties [1]. Studies conducted on the ORA showed that IOP decreased and CH increased significantly after topical PGA treatment [24, 25]. Increased CH indicates an increased corneal viscoelastic response [26]. IOP is a significant influencing factor of corneal deformation [1, 27] and should always be taken into account when analyzing corneal biomechanics. Hence, to better elucidate the corneal biomechanical changes with long-term treatment of PGA and to avoid the influences of IOP sudden decrease on it, baseline Corvis ST measurements, namely those before initiation of PGA treatment, were not analyzed.

In this study, we showed that DA, AT1 and AV1 changed with statistical significances under PGA therapy over time. Since the results were adjusted for age, gender, axial length, corneal curvature, IOP and CCT, it suggested long-term use of PGA might influence the corneal biomechanics directly. Our observation was consistent with an previous observational study, which found CH increased significantly after 6 months treatment of PGA on newly diagnosed POAG patients measured by ORA, and this increase was only significantly correlated with basal IOP but not with the drug induced IOP reduction, suggesting PGA could have a direct effect on cornea biomechanical properties [25].

A more deformable cornea is characterized by reaching the first applanation faster (with shorter AT1 and greater AV1), the second applanation slower (with longer AT2 and lower AV2) and the highest concavity with greater DA [14]. In our study, although not all of the biomechanical parameters reached statistical significances, DA (β = -0.0015), AV1 (β = -0.0004) and AT2 (β = -0.0059) decreased and AT1 (β = 0.0089) increased as PGA treatment time extended after adjusting for potential confounding factors. Based on the meanings of the parameters, our results indicated a less deformable cornea as PGA treatment prolonged.

Our observations were supported by a few previous studies as follows. Hussnain et al. observed a significant decrease in CH among medication, laser or surgery treated POAG patients in a retrospective study [28]. They hypothesized that CH decreased over time in POAG patients even though increased in a short term with therapies to reduce IOP [28]. However, the percentages and types of medical treatment were not mentioned in their publication. Another recent prospective study conducted by Meda et al. showed that POAG patients with long-term PGA therapy for at least 1 year had significantly lower CH values than those whose PGA were discontinued [12], which indicated that long-term use of PGA might cause CH decrease.

Pathogenesis differences between NTG and HTG have been investigated by many studies. Factors other than IOP probably contribute more to the onset and development of NTG. Interestingly, we found there were statistically significant differences in corneal biomechanical parameters under PGA treatment between NTG and HTG groups. After adjusting for age, gender, axial length, corneal curvature, IOP and CCT, AT1 was shorter and AV1 was greater in NTG than in HTG, which means that corneas in NTG tend to be more deformable compared to those in HTG with the extension of PGA treatment.

To our knowledge, this is the first prospective study investigating the long-term changes of corneal biomechanical properties with chronic use of PGA measured by Corvis ST. However, some of the limitations need to be considered. The patient profile may not necessarily represent the whole population data because of our clinic-based design. Furthermore, the patients had variant follow-up times and different intervals between visits, which could potentially introduce bias into current results.

## Conclusions

Our study indicated that long-term treatment of PGA might make cornea less deformable. It should be taken into consideration when analyzing corneal biomechanics in patients with PGA treatment. We speculate the corneal extracellular matrix remodeling may be involved, although the underlying mechanisms remain unknown. It would be interesting for further studies to validate these changes at a molecular level.

## Data Availability

The raw data may be made available upon reasonable request from the corresponding author. This research was approved by the Medical Ethics Committee of Eye and ENT Hospital of Fudan University (KY2012001), and adhered to the Declaration of Helsinki. Written informed consents were obtained from all of the participants.

## Abbreviations

AT1: The first applanation time
AT2: The second applanation time
AV1: The first applanation velocity
AV2: The second applanation velocity
bIOP: Biomechanically corrected IOP
CCT: Central corneal thickness
CH: Corneal hysteresis
Corvis ST: Corneal Visualization Scheimpflug Technology
DA: Deformation amplitude
HC: Highest concavity
HC: Highest concavity
HTG: High tension glaucoma
IOP: Intraocular pressure
MMPs: Matrix metalloproteinases
NTG: Normal tension glaucoma
OCT: Optical coherence tomography
ORA: Ocular Response Analyzer
PD: Peak distance
PGA: Prostaglandin analogue
POAG: Primary open angle glaucoma
SD: Standard deviation
